# Understanding the effects of PBF process parameter interplay on Ti-6Al-4V surface properties

**DOI:** 10.1371/journal.pone.0221198

**Published:** 2019-08-29

**Authors:** Trina Majumdar, Tiphaine Bazin, Emily Massahud Carvalho Ribeiro, Jessica Ellen Frith, Nick Birbilis

**Affiliations:** 1 Department of Materials Science and Engineering, Monash University, Clayton, Victoria, Australia; 2 Monash Institute of Medical Engineering (MIME), Monash University, Clayton, Victoria, Australia; 3 Ecole Nationale Supérieure de Chimie de Rennes, Rennes, France; 4 Centro Federal de Educação Tecnológica de Minas Gerais, Belo Horizonte, Brazil; University of Vigo, SPAIN

## Abstract

Ti-6Al-4V is commonly used in orthopaedic implants, and fabrication techniques such as Powder Bed Fusion (PBF) are becoming increasingly popular for the net-shape production of such implants, as PBF allows for complex customisation and minimal material wastage. Present research into PBF fabricated Ti-6Al-4V focuses on new design strategies (e.g. designing pores, struts or lattices) or mechanical property optimisation through process parameter control–however, it is pertinent to examine the effects of altering PBF process parameters on properties relating to bioactivity. Herein, changes in Ti-6Al-4V microstructure, mechanical properties and surface characteristics were examined as a result of varying PBF process parameters, with a view to understanding how to tune Ti-6Al-4V bio-activity during the fabrication stage itself. The interplay between various PBF laser scan speeds and laser powers influenced Ti-6Al-4V hardness, porosity, roughness and corrosion resistance, in a manner not clearly described by the commonly used volumetric energy density (VED) design variable. Key findings indicate that the relationships between PBF process parameters and ultimate Ti-6Al-4V properties are not straightforward as expected, and that wide ranges of porosity (0.03 ± 0.01% to 32.59 ± 2.72%) and corrosion resistance can be achieved through relatively minor changes in process parameters used–indicating volumetric energy density is a poor predictor of PBF Ti-6Al-4V properties. While variations in electrochemical behaviour with respect to the process parameters used in the PBF fabrication of Ti-6Al-4V have previously been reported, this study presents data regarding important surface characteristics over a large process window, reflecting the full capabilities of current PBF machinery.

## Introduction

Titanium-based alloys, particularly Ti-6Al-4V, are the most commonly used materials for orthopaedic implants, due to properties such as low cytotoxicity and high mechanical strength [1–4]. However, in spite of widespread clinical use, challenges remain in the use of Ti-6Al-4V for orthopaedic implants, particularly with respect to implant-bone fixation. Successful orthopaedic implant fixation requires good osseointegration, with osseointegration being defined as the formation of a direct structural and functional connection between living bone and the surface of a load-bearing implant [5–7]. Thus, for permanent fixation, the host bone must be active enough to form a stable chemical bond with the orthopaedic implant surface. Ti-6Al-4V surfaces exhibit low cytotoxicity, but unfortunately are also bio-inert, and so do not interact well with host bone cells; only weak interfacial bonds are formed following implantation, leading to inferior long term fixation [8–11]. Patients with so-called poor bone stock (bone with low bio-activity) due to pre-existing conditions (e.g. osteoporosis or atypical bone anatomy) often require implants with extra osseo-inductive features such as bone screws or surface patterning to enhance bone formation processes at the implant-bone interface [12–14]. Conventional implant manufacturing processes cannot accommodate the need for supplementary features or patient-specific implants, however, advances in additive manufacturing now allow for the production of previously un-manufacturable complex shapes, structures and features.

Additive manufacturing (AM) methods such as powder bed fusion (PBF) are becoming increasingly attractive for the fabrication of customised implants. PBF can produce highly complex components, the designs of which can be directly modelled from patient data (e.g. Magnetic Resonance Imaging or Computed Tomography scans). PBF fabrication occurs as follows; first the 3D CAD (Computer Aided Design) model is prepared and sliced into 2D layers of specified layer thickness. Within the PBF build chamber, thin layers of pre-alloyed powders are spread onto a build platform, and a high energy laser beam scans over the powder bed following the pattern of the sliced CAD model, at a set speed. The high energy laser beam melts the powder particles, forming a melt pool (also referred to as a scan track, similar to a weld track) which solidifies rapidly (<<0.1s). Once the layer is complete, the build platform is moved down by one layer and recoated with powder. The process is repeated, resulting in the layer-wise production of the component. Commonly used process parameter ranges are tabulated in Table 1.

**Table 1:** Process parameter ranges commonly used for PBF Ti-6Al-4V [15–21].

Process parameter	Common usage range
Layer thickness	20–50 μm
Particle size range	15–45 μm
Laser power	80–280 W
Laser scan speed	200–1200 mm/s
Atmosphere	Nitrogen or Argon

**Fig 1:**
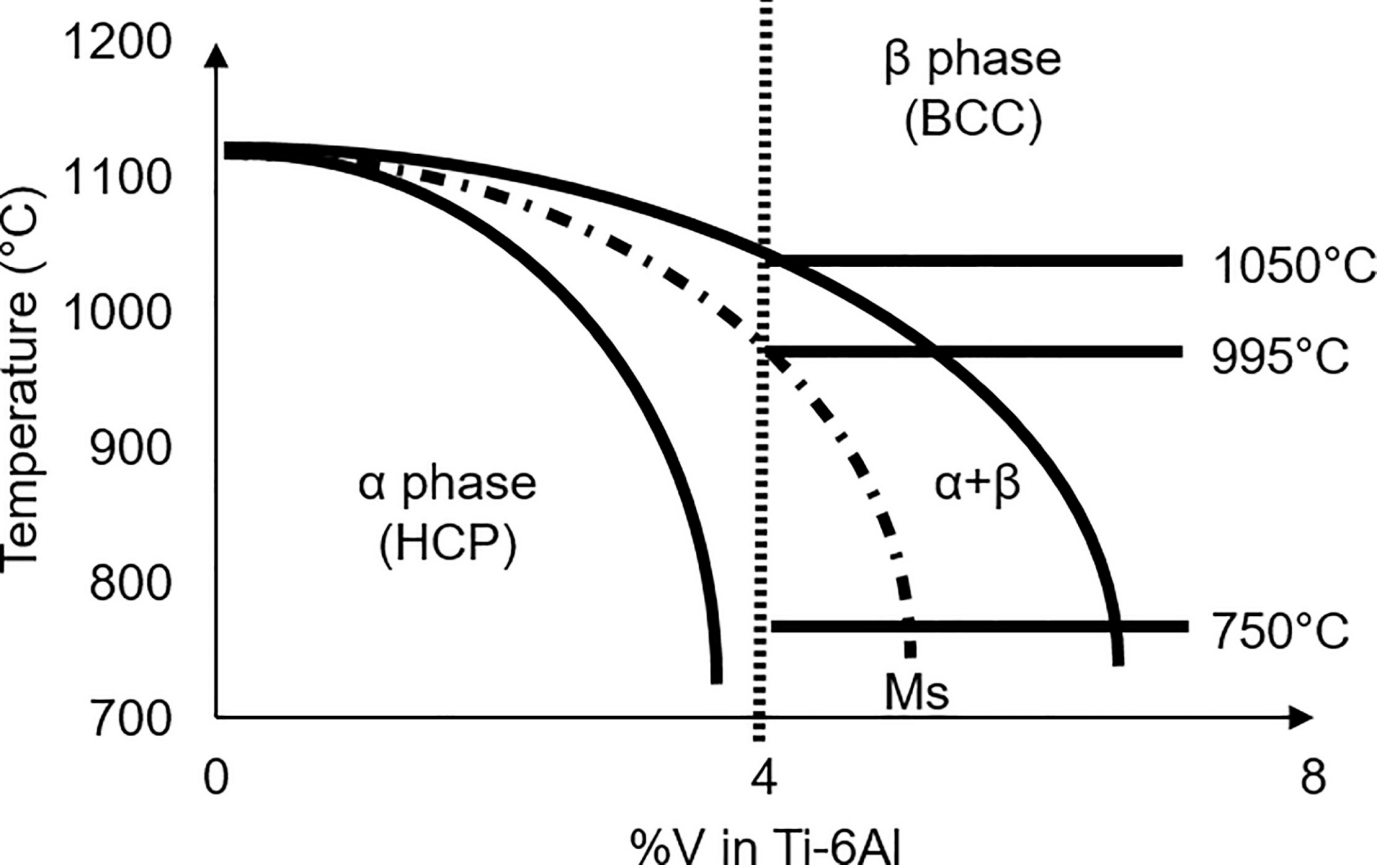
Equilibrium Ti-6Al-4V phase diagram.

PBF Ti-6Al-4V implant functionality can be modified at various stages, for example; 1) during the design stage, when features can be added into the 3D models, 2) during fabrication, by manipulating the PBF processing parameters, or 3) through various post-processing treatments. At this stage, it is clear that the choice of parameters in the PBF fabrication stage has an enormous influence on final Ti-6Al-4V properties [15,22–28], due to their effect on phase formation. Ti-6Al-4V has an allotropic temperature of approximately 995°C [29–33], with its phase diagram shown in Fig 1.

‘As-built’ PBF Ti-6Al-4V components characteristically have complex thermal histories due to the extreme heating, melting and cooling rates intrinsic to the process–which also locally vary based on component geometry. Cooling rates within the PBF melt pool can range between 12000 and 40000°C/s depending on the amount of energy supplied [34,35], and these extreme cooling rates through the transus temperature produce microstructures composed mainly of fine grained acicular α’ Ti-6Al-4V [4,35–39]. Additionally, the transfer of heat from the molten zone into the surrounding powder causes dendrite formation. The high energy laser beam, while scanning over each layer, generally penetrates through to previous layers, resulting in each layer undergoing multiple heating and cooling cycles. Therefore, fine microstructural evolution occurs during building due to the transfer of heat from the molten zone into the surrounding powder, with dendrites forming perpendicular to the applied layer, allowing vertical crystal growth through several layers [27]. These columnar β grain structures aligned along the print height can lead to anisotropy particularly with respect to tensile strength [40]. The martensitic α’ phase present in as-built PBF Ti-6Al-4V is associated with increased susceptibility to corrosion in comparison to the stable β phase partly due to a less stable TiO_2_ oxide layer [4,41], however, passive corrosion current density has been shown to increase with α’ to β phase ratio, due to the formation of a micro-galvanic cell between the two phases–which is important to consider in the choice of post heat treatments aimed at decomposition of the α’ phase into the stable α or β phases [42]. The martensitic phases and build-up of residual stresses from the layer-wise variations in cooling rates necessitate post-processing stress relieving heat treatments below the transus temperature (generally at 800°C).

Within the PBF process, many parameters can be altered, the most important of which are laser power, laser scan speed, layer thickness and hatch spacing (distance between scan lines). These first order factors affect the volumetric energy density (VED) or thermal energy supplied per unit volume, as described in Eq 1 [43–46].

$$Volumetric\;energy\;density=\frac{Laser\;power\;\left(W\right)}{laser\;scan\;speed\;\left(\frac{mm}{s}\right)\;x\;hatch\;spacing\;\left(mm\right)\;x\;layer\;thickness\;\left(mm\right)}\frac{J}{mm^{3}}$$ (1)

Certain microstructural and mechanical properties of PBF Ti-6Al-4V can be produced/tuned with careful control of the process parameters per Eq 1, and it may be possible to fabricate PBF Ti-6Al-4V implants that better support the functions and properties of natural bone than conventionally produced implants. To better utilise the potential of PBF, we must first come an understanding of how properties change with respect to process parameters chosen.

Previous work in the field includes that of Pattanayak et al [47], and Sallica-Leva et al [48] examining the PBF fabrication of Ti-6Al-4V using EOS M270 machines. Pattanayak et al examined the interplay of laser scan speed, and hatching pattern, and showed that fully dense thin plates could be obtained when maintaining a laser power to laser scan speed ratio of above 0.5, with a hatch spacing smaller than the laser diameter [47], while Sallica-Leva et al examined the relationship between energy input and porosity and determined that three main porosity levels can be produced, which are produced with changes in energy input [48]. This is in agreement with the work of Qiu et al (Concept Laser M2 Cusing), Song et al and Thijs et al. [46,49,50]. However, it should be noted that the work of Song et al and Thijs et al examine PBF Ti-6Al-4V produced with in-house constructed PBF machinery. Porosity in SLM Ti-6Al-4V has been shown to negatively affect fatigue strength (specifically micron sized pores) [35,37,51], while residual stresses have been shown to affect fatigue crack growth [33]. Shunmugavel et al further showed that the fracture mode of PBF (SLM 125^HL^) Ti-6Al-4V is transgranular due to low ductility [52]. The work of Chen et al (EOS M280, the same machinery as used in this work) indicates that apart from the process parameters, build location/direction also affect PBF Ti-6Al-4V properties [53]. Their investigations showed that while Young’s moduli did not vary across the planes, the hardness of the x-plane (perpendicular to the build plate as well as the scanning directions) was approximately 20% lower than that of the other planes, and showed reduced corrosion resistance due to high porosity, supporting the work of Murr et al [2].

In this study, Ti-6Al-4V samples were produced using 20 different combinations of laser scan speed and laser power. The properties of these samples were compared to those of samples produced using the process parameters recommended by EOS, i.e. 280 W and 1200 mm/s (referred to as EOS DEF), and wrought Ti-6Al-4V samples (referred to as WROUGHT). The range of laser scan speeds and laser powers was chosen with reference to key works in the field, as seen in Table 2. The wide range of laser powers (100–400 W) and laser scan speeds (200–1550 mm/s) used was used here to set the outer limits for process window investigations. The surfaces of the samples (parallel to the build plate) were examined with respect to their surface morphology, porosity, hardness and roughness, as well as their electrochemical behaviour in simulated body conditions. While the optimal surface parameters for osseointegration, and therefore the target properties for orthopaedic implants, are yet undefined (much controversy exists in the literature), the testing of Ti-6Al-4V surfaces produced by each PBF parameter combination will allow for the establishment of a process/property map for PBF. Current analyses of interactions between parameters have been completed for other processes and materials but little exists for Ti-6Al-4V and PBF, and not to the extent examined in this study. This data bank is necessary to inform future experiments and printing guidelines, and the future usage of PBF for orthopaedic implants and personalised medicine.

**Table 2:** Powder bed fusion process parameters used in previous studies of Ti-6Al-4V [29,33,35,49,51,54,55]. GA indicates that the powders were gas atomised, while PA indicates that the powders were plasma atomised.

Study	Machine	Laser scan speed (mm/s)	Laser power (W)	Layer thickness (μm)	Average particle size/ range (μm)
Simonelli et al., 2012 [35]	Renishaw AM250	225	157	50	15–70 (PA)
Song et al., 2012 [50]	Realiser 250	200–1600	110	30	-
Leuders et al., 2013 [33]	SLM 250HL	450	100	30	40
Qiu et al., 2013 [49]	Concept Laser M2 Cusing	800–1500	150–200	20	20–50 (GA)
Edwards and Ramulu, 2014 [51]	MTT 250	200	200	50	30 (GA)
Liu et al., 2014 [54]	SLM 250HL	710	175	30	43 (GA)
Gong et al, 2014 [56]	EOS M270	120–1560	40–160	30	25–45
Xu et al., 2015 [55]	SLM 250HL	686	375	30	15–25 (GA)
Mower and Long, 2016 [57]	EOS M280	-	-	30	15–45
Li et al, 2017 [58]	EOS M280	950–1550	150–190	30	33.77
Shang et al, 2017 [59]	EOS M280	800–1200	195	20	24–59
Ran et al, 2018 [60]	EOS M280	700	400	20	15–45

## Materials and methods

### Sample fabrication

Ti-6Al-4V samples were produced using an EOS M280 system (EOS GmbH, Germany) and pre-alloyed spherical Ti-6Al-4V powder (size range 1–53 μm (Falcontech, China)) produced through gas atomisation. A sand-blasted Ti-6Al-4V build plate was used as a substrate and pre-heated to 100°C during printing. The fabrication process took place in an argon gas atmosphere with an oxygen content of 1300 ppm, as measured prior to printing. Fixed processing parameters were as follows; hatch spacing of 0.14 mm, recoat layer thickness of 30 μm, beam offset at 0.015 mm, and stripe width at 5 mm. All samples were produced using the continuous island scan strategy. Samples were fabricated using the laser power and laser scan speed recommended by the manufacturer (EOS GmbH), and are referred to here as the EOS DEF samples. Other samples were fabricated using a range of laser scan speeds and laser powers, resulting in samples built with VED ranging from 15 J/mm^3^ to 240 J/mm^3^ (Table 3.)–each parameter combination is named MOD (for modification) followed by a number.

**Table 3:** Process parameters used to produce PBF Ti-6Al-4V specimens in this study (VED: Volumetric energy density in J/mm^3^, MOD is the naming convention given for modification).

	Laser power (W)	80	100	120	200	280
Laser scan speed (mm/s)						
**200**		**MOD 11** VED = 95	**MOD 6** VED = 120	**MOD 8** VED = 145	**MOD 5** VED = 240	**MOD 13** VED = 330
**500**		**MOD 12** VED = 40	**MOD 15** VED = 50	**MOD 9** VED = 55	**MOD 16** VED = 95	**MOD 14** VED = 135
**1000**		**MOD 18** VED = 20	**MOD 19** VED = 25	**MOD 20** VED = 30	**MOD 21** VED = 50	**MOD 1** VED = 65
**1200**		**MOD 10** VED = 15	**MOD 17** VED = 20	**MOD 7** VED = 25	**MOD 3** VED = 40	**EOS DEF** VED = 55

### Sample characterisation and testing

Following fabrication, samples were prepared as though for implantation. The samples were stress relieved by heating at 800°C for 2 hours in a vacuum furnace (cooled to room temperature in furnace), and cut from the build plate using electrical discharge machining (EDM). PBF Ti-6Al-4V is rarely used without stress relieving procedures, as the temperature gradients produced during fabrication cause significant amounts of residual stress to build up in the bulk–this necessarily causes some amount of α’ martensite phase decomposition. Wrought Ti-6Al-4V control samples were also treated at 800°C for 2 hours (referred to as WROUGHT). All samples were then ground with ethanol and water using successively finer grit silicon carbide paper (180 to 2400), and polished using Struers OP-S® and Struers MD-CHEM® cloth. All tests were conducted on sample surfaces parallel to the build plate, or the XY plane in ASTM terminology. The preparation strategy and test surfaces were chosen to approximate pre-implantation implant surfaces, however, it is important to note that as orthopaedic implants involve curvature in the x, y and z axes, it is unproductive to complete testing in all axes at this initial stage of data collection. Samples were labelled according to the parameter combination used to produce them (per Table 3.), e.g. PBF Ti-6Al-4V samples produced using a laser scan speed of 200 mm/s and a laser power of 80 W were labelled as MOD 11 samples.

Imaging of all samples was carried out using an FEI Phenom scanning electron microscope (SEM) operating at 5k V. A Bruker D8 Advance X-ray diffractometer was used to perform x-ray diffraction (XRD) on samples. A Cu Kα radiation source was used, with analysis performed at 40 kV and 40 mA. The diffraction angle range used was between 30° and 100°, with a step increment of 0.5° and a time step of 0.25 seconds. The obtained spectra were compared to reference data from the International Centre for Diffraction Data using DIFFRACplus EVA software (AXS, Bruker, Karlsruhe, Germany).

Average surface roughness (Ra) was analysed using a Veeco Wyko NT1100 optical profilometer. The samples were imaged using vertical shift interference (VSI) mode, 1.0x magnification, 1.0x laser scan speed and a threshold of 0.1%. The back scan and the length were set at 200 μm and 500 μm. 5 measurements were taken for each sample group, with re-grinding and polishing steps following each measurement (i.e. multiple layers were examined).

Porosity was determined using optical microscopy, rather than porosimetry techniques, due to the nature of the porosity expected; the non-connected (and stochastic) pores are likely to contain unmelted or partially melted particles, as there is no scope for particle removal during or following the formation of these pores. The agglomeration of particles within these pores affects the measurement of density and porosity through displacement techniques or porosimetry respectively, and thus porosity was determined through optical analyses. All samples were sonicated for 3 minutes in ethanol, dried with compressed air, and imaged using an Olympus GX51 optical microscope at 5x magnification. Image analysis (to determine % porosity and average pore area) was carried out using ImageJ (v1.50b), an image analysis software created by Wayne Rasband of the National Institutes of Health (Bethesda, MD, USA)–this software is commonly used in biomaterials research. Each image was opened in ImageJ, split according to colour channel, then manually thresholded. The ‘Analyse Particles’ feature of ImageJ was then used to count and size the pores (in terms of 2D area), with a minimum pore area set as 10 μm^2^, and a minimum circularity set as 0.5 (i.e. more than 50% circular). Three images (and measurements) were taken for each category, with re-grinding and polishing steps following each measurement. Sample hardness was measured using a Wilson Rockwell hardness tester. A 120° diamond spheroconical indenter (C Rockwell scale) was used with a load of 150 kg. 10 measurements were made for each category.

Electrochemical testing was carried out in a double-chambered three-electrode electrochemical cell connected to a Biologic VMP-3Z potentiostat. All samples were mounted in epoxy, with the exposed surface acting as the working electrode, with copper tape or wires extending from the rear to allow voltage measurement. A platinum mesh was used as the counter electrode, and a saturated calomel reference electrode was placed in a Haber-Luggin capillary extending to within 5 mm of the sample surface. A biologically relevant media was used as the electrolyte to approximate in vivo conditions—minimum essential media (MEM) was made up from powder (Gibco GlutaMAX) (1 packet added to 1L of water with 2.2 g of sodium bicarbonate), and the pH of this solution was adjusted with 5% HCl/1M NaOH to pH 7.2 ± 0.2. The composition and ion concentrations present in this solution can be seen in S1 Table. The electrolyte was maintained at 37°C using a circulating water bath. All samples were left to rest for 10 minutes minimum at open circuit potential (OCP), following which potentiodynamic polarisation was carried out from– 150 mV / Eoc to 1.0 V / Eref (reference electrode potential) with a scan rate of 1 mV/s.

## Results

### Morphology and porosity of as-produced and polished PBF Ti-6Al-4V surfaces

XRD analysis of samples as compared to references from the International Centre for Diffraction Data (PDF Refs. 01-072-5007, 00-044-1294, 00-022-1058 and 00-044-1288) indicated that all samples displayed evidence of α phase Ti peaks and minimal evidence of β phase Ti peaks (S1 Fig). Each process parameter combination produced samples with majority HCP α phase Ti-6Al-4V. Immediately following building, PBF Ti-6Al-4V is composed predominantly of acicular α’ phase [49,61–63], but following stress relieving procedures, a significant portion of this metastable α’ phase decomposes into the stable α phase.

**Fig 2:**
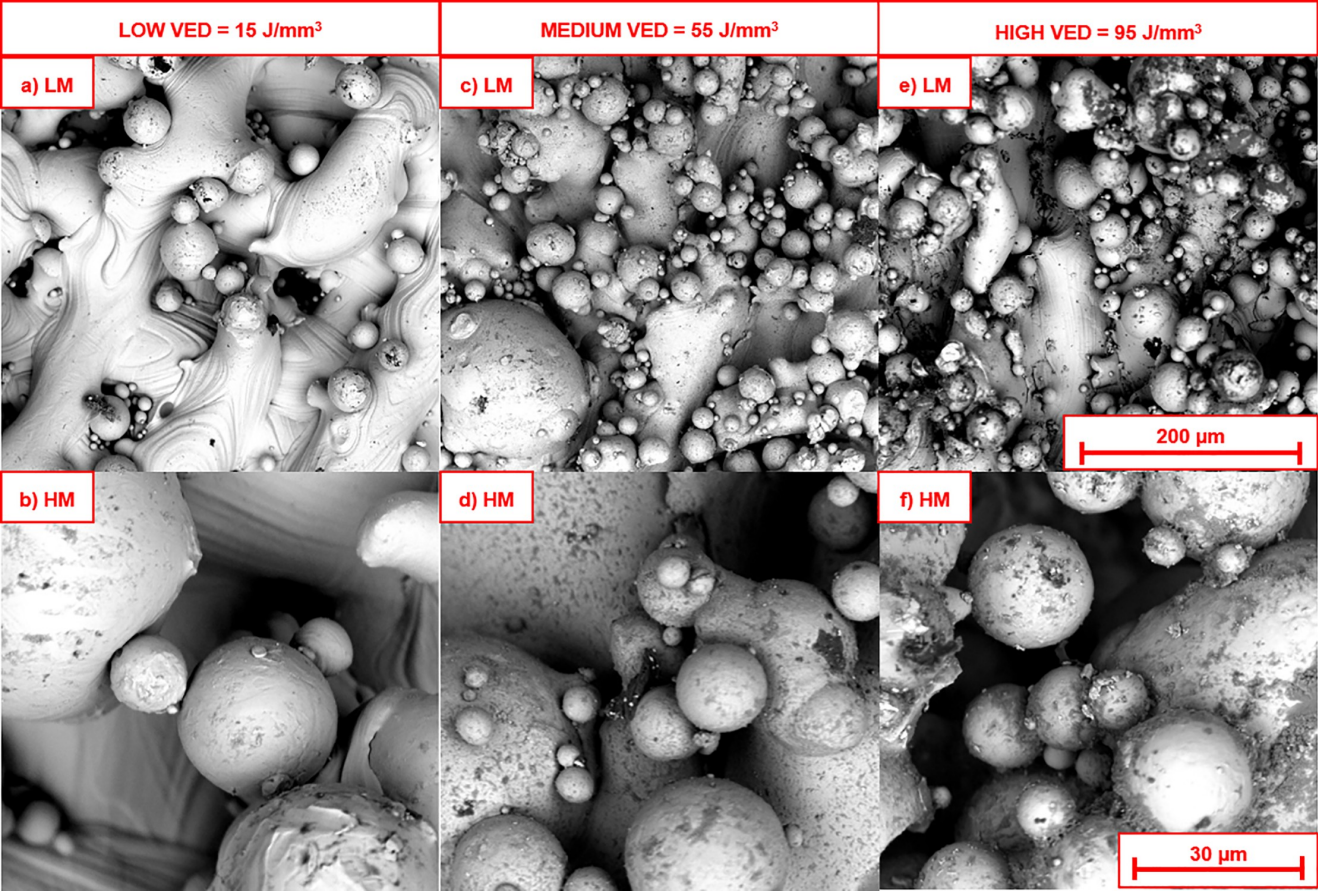
Scanning electron micrographs of the as-produced surfaces, showing: a, b) Specimen prepared with low energy density (MOD 10: 80 W, 1200 mm/s, VED = 15 J/mm^3^) at low and high magnification, c, d) Specimen prepared with intermediate energy density (EOS DEF: 280 W, 1200 mm/s, VED = 55 J/mm^3^) at low and high magnification, e, f) Specimen prepared with high energy density (MOD 16: 200 W, 500 mm/s, VED = 95 J/mm^3^) at low and high magnification.

The uppermost layers of all of the stress relieved, unpolished surfaces were covered in partially melted particles, as seen in Fig 2. As previously mentioned, the outer layers of PBF Ti-6Al-4V experience different cooling rates to the bulk, due to fewer re-melting/re-cooling cycles caused by laser penetration. Heat energy dissipates readily into the surrounding powder, leaving partly melted/fused particles across the layers. Notably, more partly fused particles were observed along the z-plane edges of PBF Ti-6Al-4V, as compared to the XY topmost layer (parallel to the build plate). The amount of partially fused particles on the outer layers are also influenced by powder splash caused by the laser beam diameter (spot size) and hatch spacing used. The samples produced with low VED (< 50 J/mm^3^) had comparatively fewer partially melted particles across their x/y surfaces than those produced with higher VED (> 55 J/mm^3^), likely due to comparatively exaggerated cooling rates/heat transfer between the final layer and the surrounding powder caused by the higher thermal energy supplied per unit volume. The production of rough outer surfaces is near unavoidable with PBF, and thus these outermost layers are generally removed prior to usage. As-built surface roughness, however, is important to consider with respect to the future reduction of post-processing treatments.

**Fig 3:**
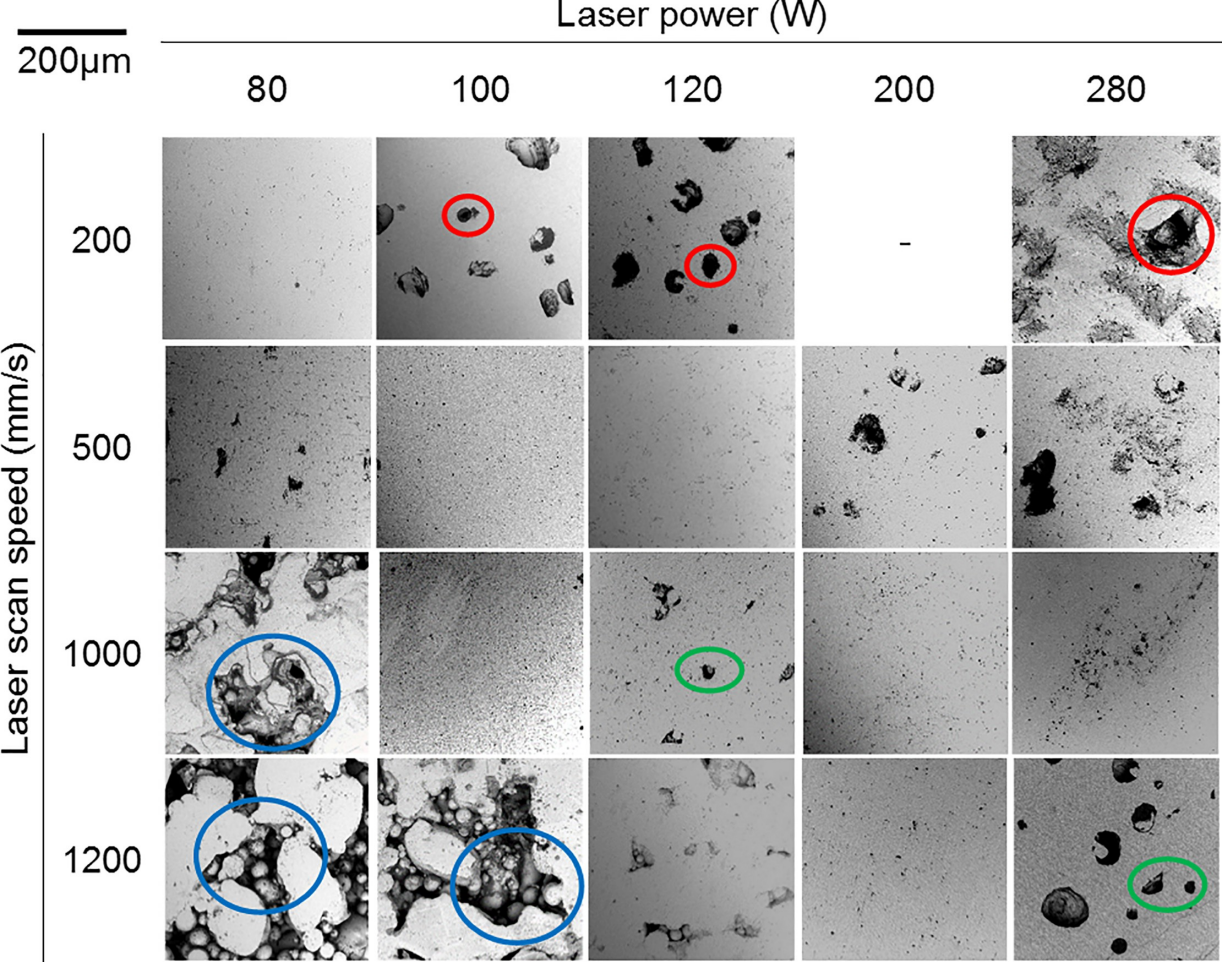
Secondary electron micrographs of polished surfaces corresponding to various laser parameter combinations. Burn-through pores circled in red, lack of fusion pores (with partially melted particles) circled in blue, and near-spherical pores circled in green.

Following the grinding back and polishing of the uppermost XY layer of the samples, distinct variations in surface appearances could be observed even without etching. Black dots were seen on the majority of sample surfaces produced by each process parameter combination, as seen in Fig 3, indicating the presence of pores formed through either gas entrapment or accumulated layer-wise defects produced during fabrication.

The sample surfaces in the top left quadrant of Fig 3., i.e. those produced with low laser power/low laser scan speed exhibited irregular distributions of pores and defects. The combination of high laser scan speed and low laser power have the layer-wise effect of providing insufficient energy to fully melt all particles, leaving partially melted particles across each layer, affecting the deposition of subsequent layers. Insufficient powder consolidation may be partly fixed through the use of multiple laser passes per layer (increases costs), or avoided through the careful balancing of process parameters in the first instance.

**Fig 4:**
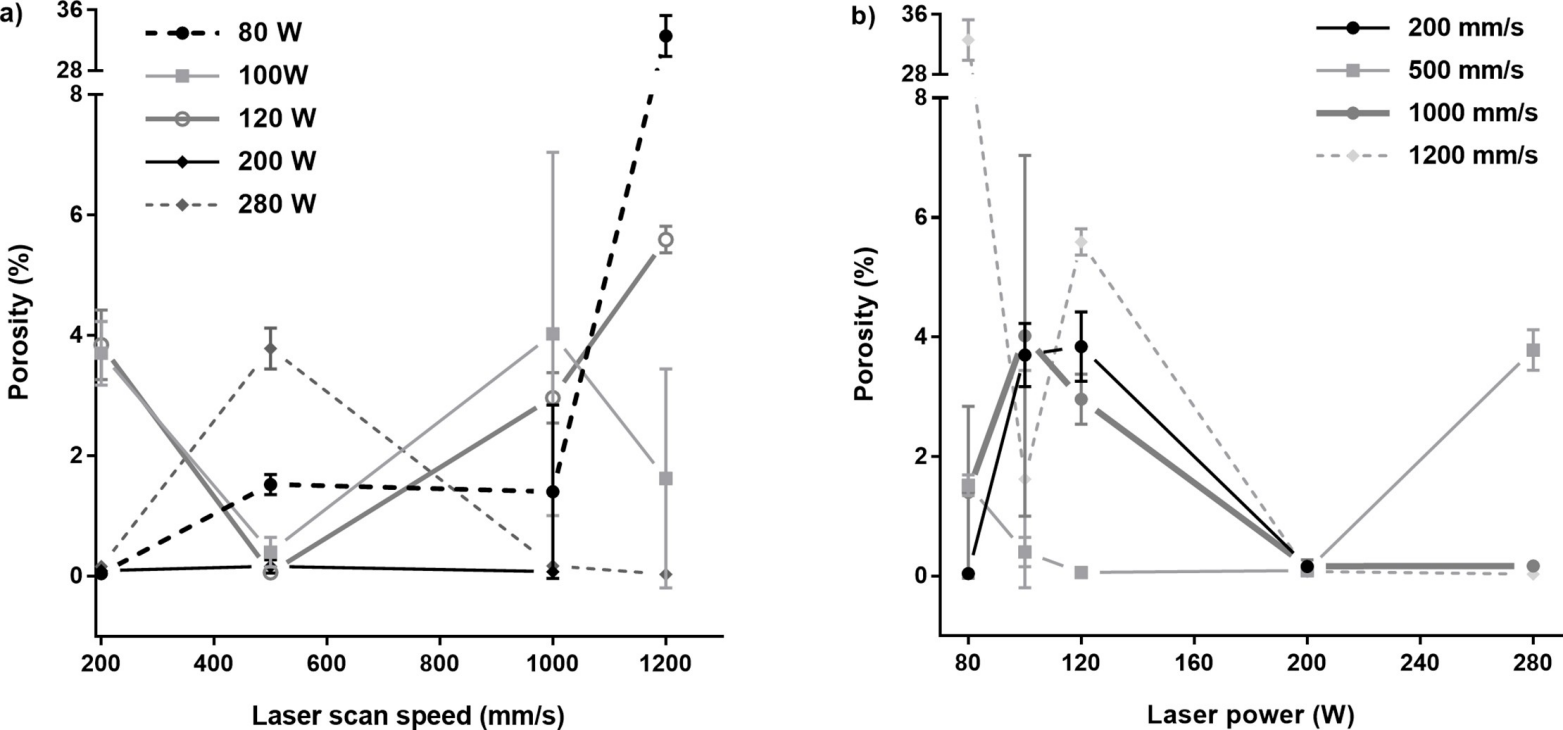
Average porosity (in %) produced as a function of PBF parameter combinations, a) showing porosity as a function of laser scan speed, b) showing porosity as a function of laser power.

Increasing laser scan speed from 200 mm/s to 1200 mm/s while keeping laser power constant (i.e. top to bottom on Fig 3.) affected powder consolidation. At low laser power (80W), increasing laser scan speed was accompanied by larger proportions of incompletely melted particles scattered throughout build height, as well as large gaps where completely fused layers were not able to form. At intermediate and high laser powers (100-280W), increases in laser scan speed had a roughly parabolic effect on the quantity of manufacturing defects. At low laser scan speeds (200–500 mm/s), laser powers of 100–200 W resulted in the supply of excessive local thermal energy, causing the formation of keyhole pores within the samples. At higher laser scan speeds (> 500 mm/s), the energy supplied was more evenly spread, reducing the number of keyhole pores, however the number of lack of fusion (LoF) and metallurgical pores increased. LoF and metallurgical pores form due to fast cooling, preventing gas escape from the melt pool. At the upper limits of laser scan speed (1200 mm/s), porosity again increased–the combination of intermediate laser power with high laser scan speed resulted in large sections of incompletely melted particles. At high laser power (280 W), high laser scan speeds produced samples with large quantities of keyhole pores as opposed to metallurgical pores. This is visualised graphically in Fig 4.

**Fig 5:**
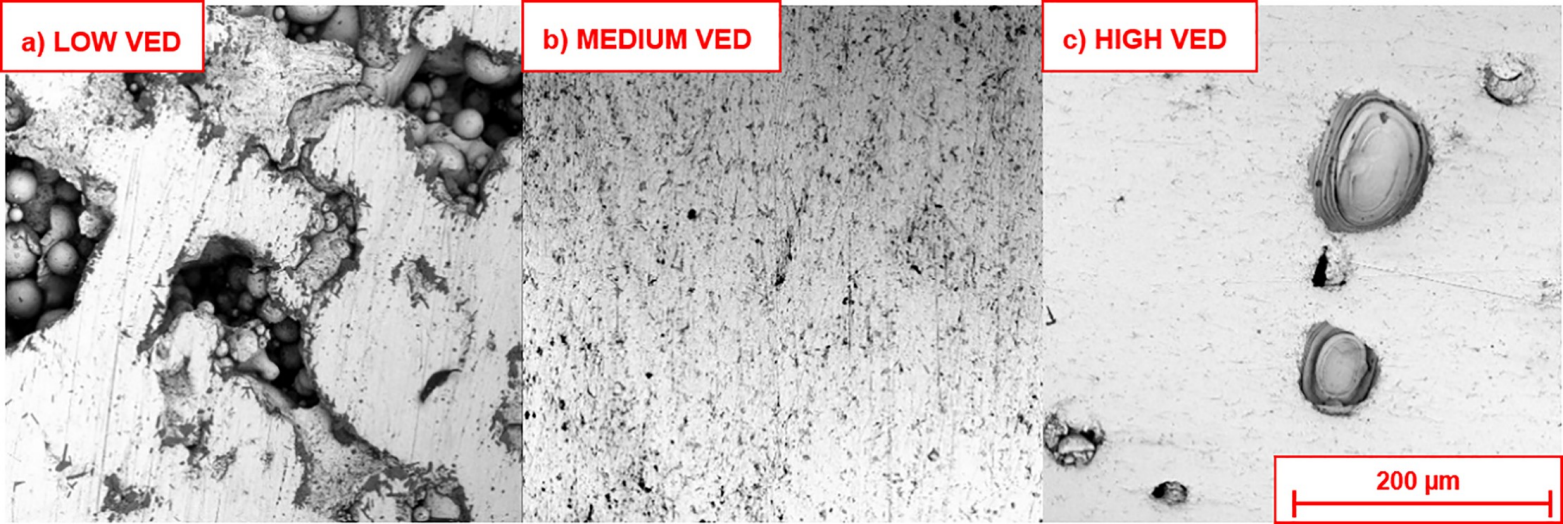
Secondary electron images of pore type with respect to energy density range. a) representative pores produced at low energy density (80 W, 1200 mm/s, VED = 15 J/mm^3^), b) representative pores produced at intermediate energy density (280 W, 1200 mm/s, VED = 55 J/mm^3^) and c) pores produced at high energy density (200 W, 500 mm/s, VED = 95 J/mm^3^).

The EOS DEF settings (80 W, 1200 mm/s, VED = 55 J/mm^3^) produced samples with both the lowest percent porosity and the smallest average pore areas, of 0.03 ±0.01% and 43.8 ± 4.1 μm^2^ respectively. The largest average pore areas and percent porosity of 3383.6 ± 246.6 μm^2^ and 32.59 ± 2.72% were produced with the MOD 10 settings (280 W, 1200 mm/s, VED = 15 J/mm^3^). Interestingly, both EOS DEF and MOD 10 samples were produced using a laser scan speed of 1200 mm/s but with the highest and lowest laser power respectively (80 and 280 W). Though pore shape irregularity impedes the precise quantitative comparison of pore area, a qualitative comparison can be made between the pores produced using each combination. Porosity obtained in samples formed with different VEDs can be seen in Fig 5.

**Fig 6:**
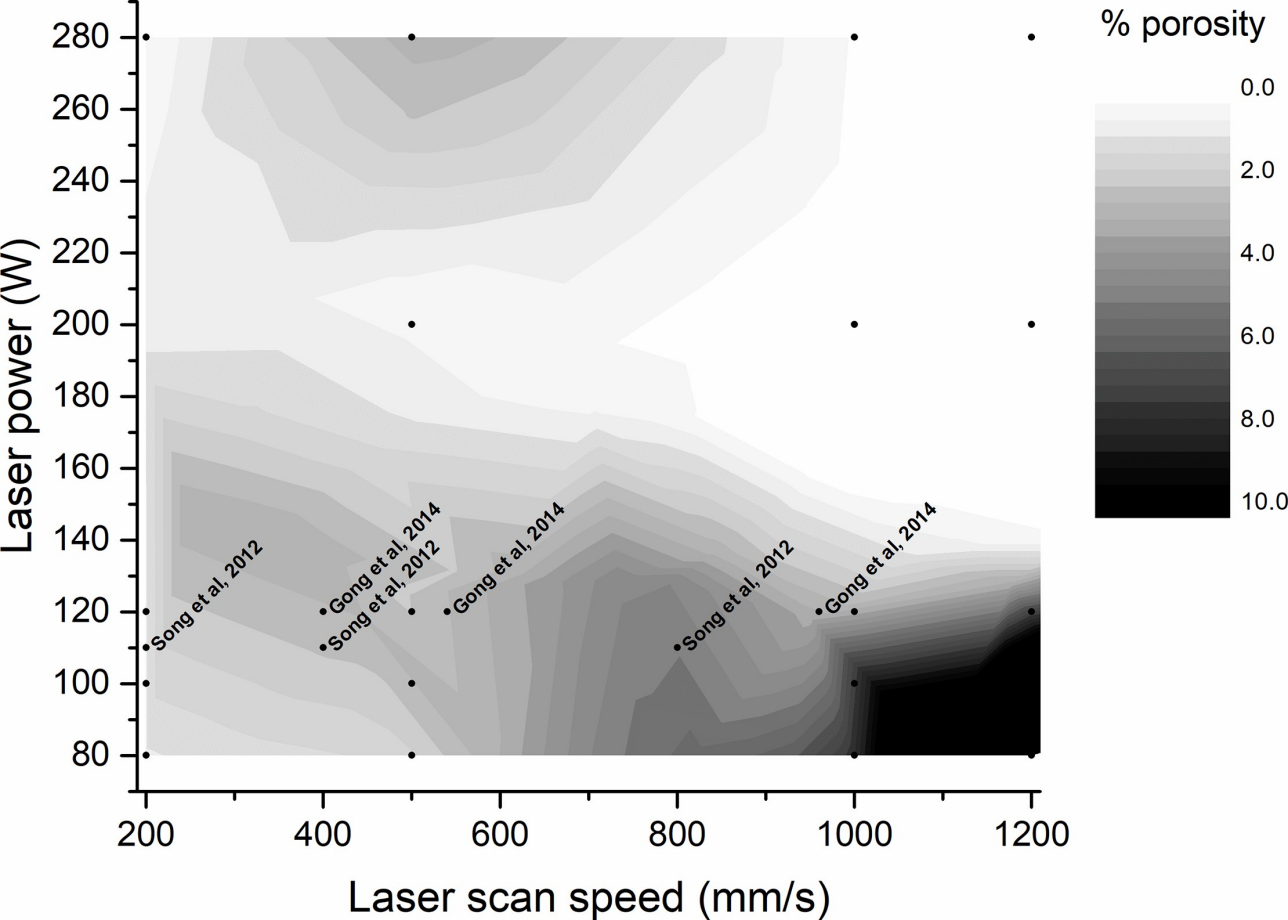
Porosity contour map for PBF Ti-6Al-4V with respect to laser power and laser scan speed.

As expected, the % porosity produced using each parameter combination was somewhat linked to calculated VED. Samples produced using low VEDs, 15 to 40 J/mm^3^, exhibited large and irregular pores, as well as incompletely melted particles (average porosity percentage orange between 0.03 ±0.01% and 32.59 ± 2.72%). The highest VEDs, 70 to 140 J/mm^3^, formed samples with small, regular pores with very few unmelted particles (average porosity percentage between 0.04 ± 0.01% and 3.70 ± 0.53%). The lowest porosity was obtained using intermediate VEDs, between 40 and 60 J/mm^3^ (average porosity percentage between 0.03 ± 0.01% and 0.40 ± 0.25%). This is in direct opposition to previous work by Bertoli et al [43], El Sayed et al [15] and Song et al [26], who state that ideal porosity is produced when the VED is maintained at above 100 J/mm^3^, between 90–130 J/mm^3^ and between 120–140 J/mm^3^ respectively. A contour map of porosity arising from the combination of various laser scan speeds and laser powers can be seen in Fig 6, alongside data from Gong et al [56] and Song et al [50].

While a general relationship was observed between calculated VED and porosity percentage, it is important to note that the individual parameters contributing to VED i.e. laser power and laser scan speed, may have a larger influence on final porosity. Samples built using the same VED but with differing parameters (i.e. different laser scan speed and laser power resulting in the same VED) displayed different porosity levels, indicating that the interplay of the process parameters had a larger influence on porosity than the calculated VED value itself. Specifically, the parameter combinations used to build MOD 12 (80 W, 500 mm/s) and MOD 3 (200 W, 1200 mm/s) supplied the same VED of 40 J/mm^3^, but these samples displayed differing porosity percentages of 1.52 ± 0.17% and 0.07 ± 0.01% respectively.

### Variations in roughness and hardness of PBF Ti-6Al-4V according to process parameter interplay

This study examined the PBF fabrication of Ti-6Al-4V with a view to ultimately producing customised orthopaedic implants for patients. Thus, all samples were polished as per guidelines for implant materials (ASTM F1108b). However, significant differences in roughness were observed even following polishing. Layer-wise balling can affect subsequent layer re-coating and thus affect the bulk and final surface topography.

A large range of surface roughness was produced within the experimental process window, with average roughness, R_a_, ranging from 0.40 ± 0.01 μm (MOD 3: 200 W, 1200 mm/s, VED = 40 J/mm^3^) to 17.97 ± 4.10 μm (MOD 10: 80 W, 1200 mm/s, VED = 15 J/mm^3^). Layer wise roughness arises in PBF Ti-6Al-4V due to both balling and incomplete particle fusion. Neither roughness nor hardness showed a significant relationship to VED as per Fig 6, in agreement with the work of Song et al [50] and Kruth et al [64] who reported balling at both high and low VED respectively. Rather, the combination of laser scan speed and laser power had a greater influence on roughness and hardness. The EOS DEF settings (280 W, 1200 mm/s, VED = 55 J/mm^3^) produced surfaces with an R_a_ of 4.62 ± 0.9 μm.

**Fig 7:**
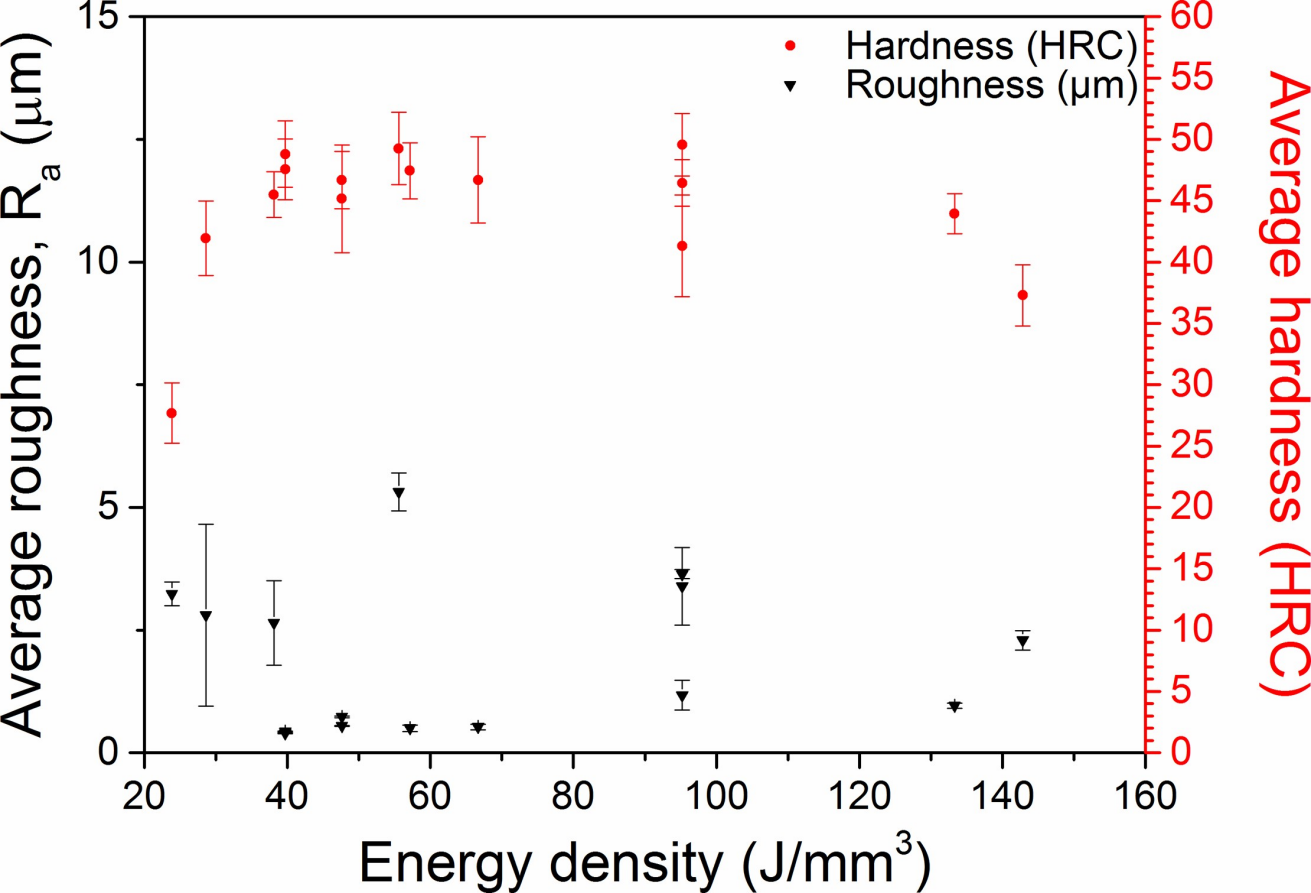
Average Rockwell Hardness (HRC) and roughness (R_a_) as a function of VED used to prepare specimens.

The experimental process window produced samples with average hardness ranging from 35.6 ± 2.0 HRC (MOD 7: 120 W, 1200 mm/s, VED = 25 J/mm^3^) to 49.0 ± 2.9 HRC (MOD 6: 100 W, 200 mm/s, VED = 96 J/mm^3^), with the EOS DEF settings (280 W, 1200 mm/s, VED = 55 J/mm^3^) producing samples with a hardness of 48.7 ± 3.2 HRC, as compared to WROUGHT with a hardness of 36 HRC [27,63,65], as seen in Fig 7 (note that porosity above 10% prevents the accurate determination of hardness values and thus certain data sets were omitted). The high hardness of PBF Ti-6Al-4V arises due to the rapid cooling rate of the process. High hardness α’ martensite nucleates at prior β grain boundaries and grows within parent β grains [61,65,66]. Post-process stress relieving heat treatments have been shown to facilitate the decomposition of the α’ phase into lamellar α+β microstructures through α precipitation at the α’ boundaries [29,67]. According to the XRD data (S1 Fig), low intensity β peaks could be observed in all samples, indicating that while α’ phase decomposition occurred during the stress relief treatment, the proportion of β phase is likely to be low. Certain parameter combinations produced samples with similar hardness to that of wrought Ti-6Al-4V; MODs 7 and 8 (MOD 7: 120 W, 1200 mm/s, VED = 25 J/mm^3^, MOD 8: 120 W, 200 mm/s, VED = 145 J/mm^3^) exhibited hardness values of 35.6 ± 2.0 and 36.6 ± 2.9 HRC respectively, despite having been fabricated with the highest and lowest laser scan speeds in the process window.

The relationship between surface roughness and hardness has not been extensively investigated with regards to PBF Ti-6Al-4V and bears further inquiry.

### The effects of PBF process parameter interplay on corrosion resistance and repassivation characteristics

The corrosion behaviour of PBF Ti-6Al-4V has not received much attention in comparison to properties such as fatigue resistance or elastic modulus, however, understanding the corrosion characteristics of materials used in orthopaedic implants is of paramount importance. To date, relatively few groups have systematically examined the corrosion behaviour of stress relieved PBF Ti-6Al-4V under simulated physiological conditions. Dai et al report that as-built PBF Ti-6Al-4V exhibits reduced corrosion resistance as compared to wrought Ti-6Al-4V due to the majority α’ microstructure [68], while Yang et al report that the inferior corrosion resistance of as-built PBF Ti-6Al-4V can be corrected through an appropriate heat treatment, i.e. holding at a sub-transus temperature for 2 hours [69]. The electrochemical behaviour of the samples examined in this study, having undergone an appropriate heat treatment as recommended by Yang et al (i.e. held at 800°C for 2 hours), are thereby appropriate for use in predicting behaviour in the human body. More recently, Chiu et al studied the electrochemical behaviour of PBF Ti-6Al-4V formed with laser powers between 42 and 50 W and laser scan speeds between 100 and 400 mm/s, and indicate that the surface titanium oxide layer acts as an n-type semiconductor [70]. However, all of these studies were conducted in simple solutions such as NaCl or Ringer’s solution, and thus fail to incorporate the effects of other compounds found in human body fluids such as amino acids and vitamins.

In this study, changing the laser scan speed and/or laser power used in sample fabrication had a pronounced effect on electrochemical behaviour, even considering that specimens tested underwent stress relief heat treatment (Table 4.). Electrochemical testing was completed on multiple samples in simulated physiological conditions, i.e. at 37°C in MEM. The scatter in the current densities observed between samples of the same category indicate that factors apart from the laser scan speed and laser power affect electrochemical behaviour. It has been reported that PBF component properties such as porosity and roughness can vary spatially across the build plate [71], and thus the scatter in data is important to consider in any recommendations as to ranking parameter combinations.

**Table 4:** Corrosion properties of PBF Ti-6Al-4V produced with varied process parameters, as determined from potentiodynamic polarisation curves.

Specimen ID	Laser scan speed (mm/s)	Laser power (W)	VED (J/mm^3^)	E_corr_ (mV_SCE_)	i_corr_ (μA/cm^2^)	Porosity (%)
WROUGHT	n/a	n/a	n/a	-451 ± 70	0.07 ± 0.03	n/a
EOS DEF	1200	280	55	-412 ± 23	0.10 ± 0.05	0.03 ± 0.01
MOD 1	1000	280	65	-348 ± 39	0.07 ± 0.06	0.17 ± 0.08
MOD 3	1200	200	40	-398 ± 76	0.13 ± 0.07	0.07 ± 0.01
MOD 6	200	100	95	-450 ± 64	0.13 ± 0.06	3.70 ± 0.53
MOD 7	1200	120	25	-158 ± 29	1.06 ± 0.14	5.59 ± 0.22
MOD 8	200	120	145	-473 ± 41	0.15 ± 0.07	3.84 ± 0.58
MOD 9	500	120	55	-615 ± 60	0.12 ± 0.08	0.06 ± 0.01
MOD 10	1200	80	15	-387 ± 30	2.32 ± 1.79	32.59 ± 2.72
MOD 11	200	80	95	-603 ± 42	0.09 ± 0.02	0.04 ± 0.01
MOD 12	500	80	40	-497 ± 102	0.07 ± 0.02	1.52 ± 0.17
MOD 13	200	280	335	-567 ± 57	0.08 ± 0.05	0.16 ± 0.04
MOD 14	500	280	135	-572 ± 16	0.10 ± 0.06	3.78 ± 0.34
MOD 15	500	100	50	-578 ± 31	0.04 ± 0.01	0.40 ± 0.25
MOD 16	500	200	95	-532 ± 40	0.08 ± 0.06	0.09 ± 0.04
MOD 17	1200	100	20	-430 ± 28	1.37 ± 0.35	1.62 ± 1.82
MOD 18	1000	80	20	-355 ± 26	2.71 ± 0.41	1.40 ± 1.44
MOD 19	1000	100	25	-296 ± 32	0.54 ± 0.09	4.02 ± 3.02
MOD 20	1000	120	30	-466 ± 24	0.15 ± 0.04	2.96 ± 0.42
MOD 21	1000	200	50	-503 ± 43	0.10 ± 0.05	0.16 ± 0.11

**Fig 8:**
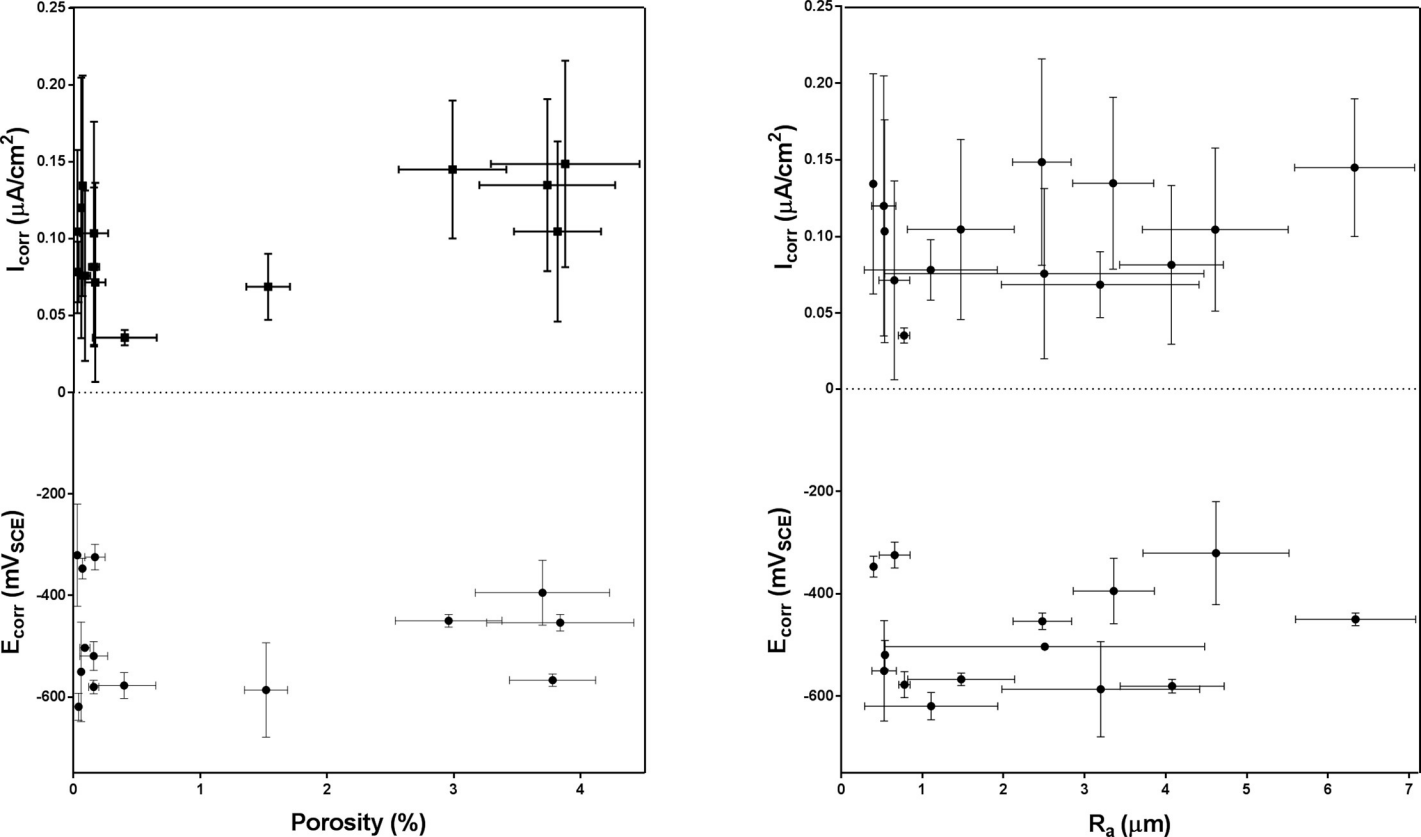
Porosity and E_corr_ did not display a straightforward relationship, due to the presence of both partially melted particles within pores and alterations in surface chemistry through keyholing.

As Ti-6Al-4V is a relatively inert alloy in terms of corrosion, the large variation in E_corr_ according to process parameters used is unusual and bears detailed examination. The samples with the lowest corrosion resistance of -615 ± 60 mV_SCE_ (MOD 9: 120 W, 500 mm/s, VED = 55 J/mm^3^) were fabricated using a well-balanced combination of laser power and laser scan speed, and the calculated VED equalled that of the EOS DEF settings. The current density for these samples was three times that of the samples with the lowest current densities of 0.04 ± 0.01 μA/cm^2^ (MOD 15: 100 W, 500 mm/s, VED = 50 J/mm^3^). Interestingly, the MOD 9 samples, while showing the highest corrosion rate, were found to have among the lowest % porosity (0.06 ± 0.01%) and R_a (_0.53 ± 0.15 μm). This is visualised in Fig 8.

**Fig 9:**
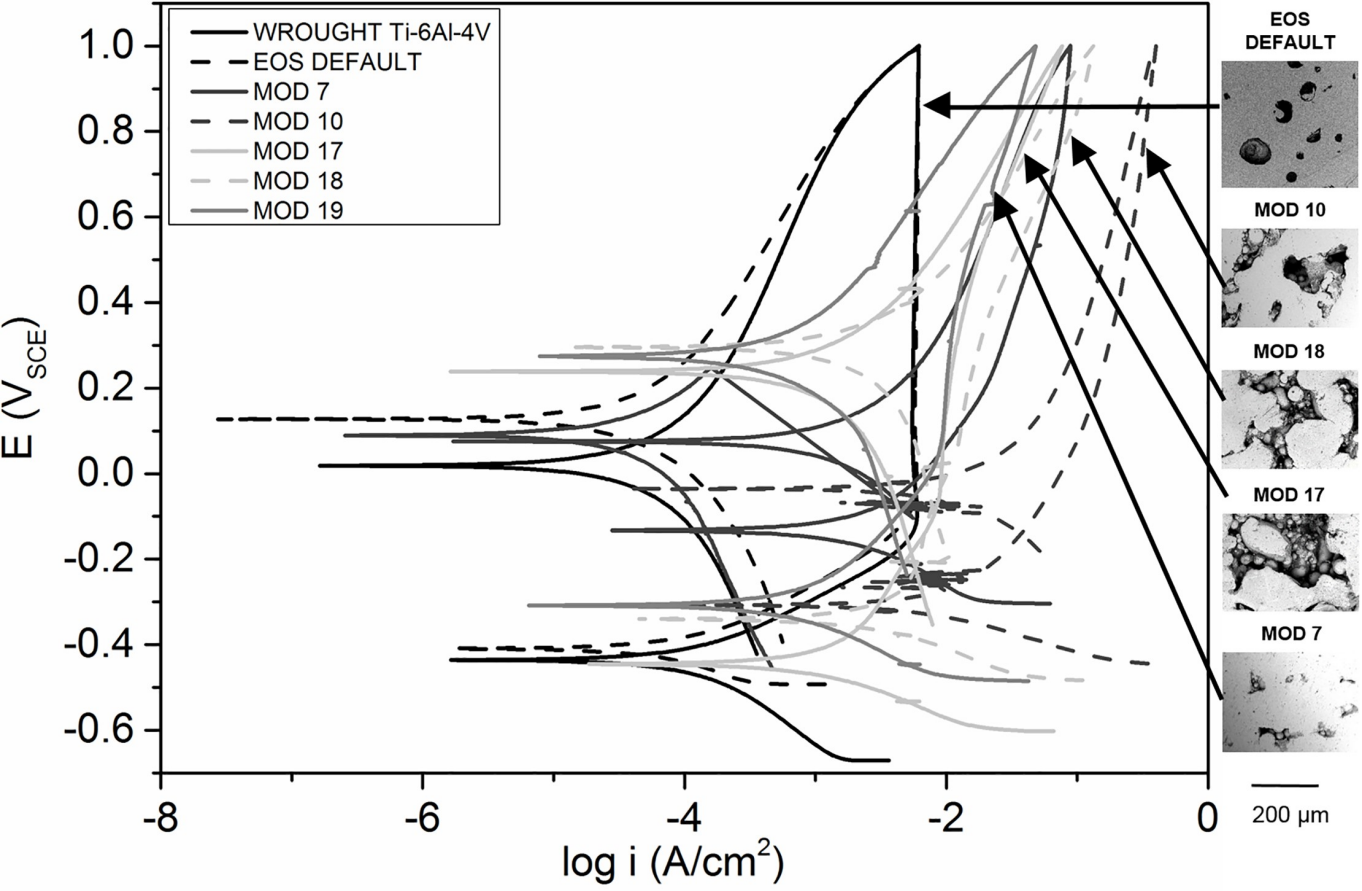
Representative cyclic potentiodynamic polarisation curves for specimens produced using various PBF parameter combinations, showing extremely altered repassivation behaviour.

Of the PBF Ti-6Al-4V samples, 5 sample groups were notable for displaying drastically different electrochemical behaviour, with regard to the shape of the re-passivation window; these were produced with parameter combinations in the bottom left quadrant of the parameter combination matrix, indicating a combination of low laser scan speed and laser power, and energy densities below 30 J/mm^3^. It can be inferred that the heterogeneity in surface morphology (particularly porosity) caused by incomplete particle melting due to low energy input leads to poor corrosion resistance and re-passivation, and may affect the formation of the surface oxide. Each group had similar I_corr_ values, but significantly different E_corr_ values–these are visualised in Fig 9. MOD 10 (80 W, 1200 mm/s), with the lowest VED of 15 J/mm^3^, displayed the highest corrosion current density of 2.32 ± 1.79 μA/cm^2^. MOD 7 (120 W, 1200 mm/s, VED = 25 J/mm^3^) showed the least active E_corr_ of -158 ± 29 mV_SCE_ Comparatively, MOD 12 (80 W, 500 mm/s) with a slightly higher VED of 40 J/mm^3^, showed a ‘normal’ re-passivation window, with an E_corr_ of -497 ± 102 mV_SCE_, in the range of wrought Ti-6Al-4V.

This analysis however assumes that the active area of all the samples is the same–realistically the surface area due to pores may be much larger, and so the actual current density (as opposed to calculated current density) may be lower. The samples with the highest porosity (MODs 7, 10, 17, 18, 19) did display the highest current densities, as well as reduced ability to re-passivate, but appeared to have higher corrosion resistance than most of the other samples. The measurement of active surface area was out of the scope of this study, but it is clear that apparent current density (and apparent surface area) is more relevant when it comes to predicting Ti-6Al-4V implant behaviour in the body. Non-stochastic corrosion initiation is likely to have occurred, with pores acting as pre-existing pits, leading to crevice corrosion-like processes and random spikes in current density according to the pores ‘activated’. However, no severe pitting was observed, even up to 2 mV_SCE_, and so the actual likelihood of pitting corrosion in the body is low, even for the most active samples examined.

## Discussion

**Fig 10:**
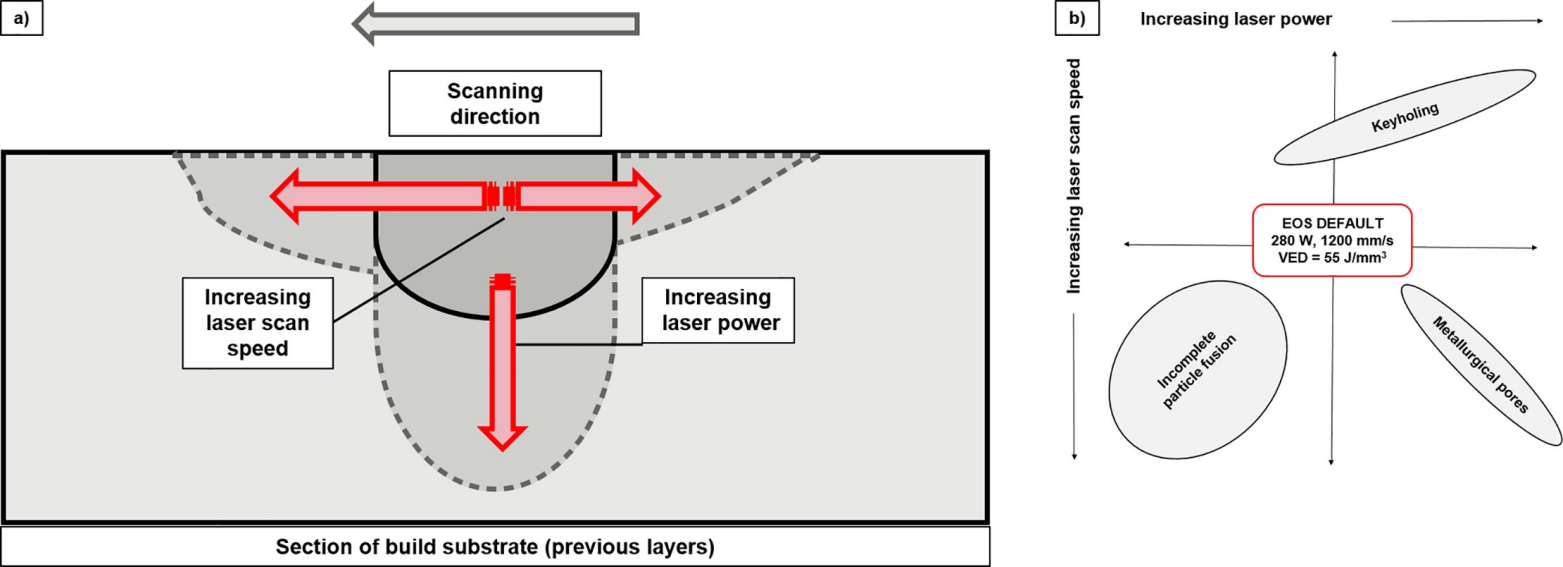
Changing laser scan speed or laser power affects melt pool depth during the PBF build process, with a phenomenological diagram shown in a) and a graphical schematic of the specific defects formed shown in b).

To explain the generation of layer-wise roughness and porosity in PBF, it is necessary to understand the behaviour of the moving melt pool [17,25,43,64,72,73]. As the laser beam interacts with the powder bed, a pool of molten Ti-6Al-4V is formed, and as the laser beam scans across the bed, the melt pool cools to form a weld track. The cooling melt pool/weld track is subject to Plateau-Rayleigh instability due to perturbations arising from the extreme cooling rates of the melt pool–as the track cools, the melt pool is driven to break up into smaller droplets to reduce its surface energy and area. Should the level of this instability be too high, distinct ‘balls’ of Ti-6Al-4V will appear along the weld tracks [17,26,73]. Within the moving melt pool, both surface tension and temperature gradients form, resulting in thermo-capillary convection flow (the Marangoni effect). Balling and partially melted particles can affect subsequent layer deposition and adherence. When subsequent layers are melted onto previous layers, there is potential for partially melted particles to be re-melted due to the multiple layer penetration of the laser, however, as the bulk forms, a random distribution of partially melted particles and pores will form throughout the build height. Once the track has solidified, small wavelike ripples are apparent on the weld tracks [73], as well as partially melted particles. Therefore, surface topographies and indeed porosity are unavoidable without the careful choice of processing parameters to maintain the dynamic equilibrium of the melt pool. This is shown schematically in Fig 10. Laser power and laser scan speed have the greatest effect on the melt pool, with increases in scan speed causing the size of the melt pool to increase, while increasing scan speed elongates/narrows and lowers the temperature of the melt pool [23]. Increasing the overall VED is linked to comparatively more gradual cooling rates.

Currently, there is little consensus in the biomedical community on the optimal porosity/roughness for osseointegration [74–76]. The optimal roughness values for osseointegration have variously been reported as R_a_ > 1 μm, R_a_ = 3 to 5 μm and even R_a_ > 5 μm [6,77–79]. With respect to the results of this study, it is clear that any of these roughness values can be achieved. With this understanding of how surface roughness, porosity, hardness and electrochemical behaviour changes with changes in laser scan speed and laser power, it is possible to choose the most appropriate laser scan speed and laser power to produce PBF Ti-6Al-4V components with the required properties. However it should be noted that porosity and roughness have a significant effect on wear and fatigue performance and thus future work should explore the changes in fatigue behaviour between Ti-6Al-4V built with various process parameter combinations. In the current study, each of the non-standard process parameter combinations used produced samples with one or more improved properties when compared to wrought Ti-6Al-4V, and so it is clear that sample properties can be tuned through careful manipulation of the PBF process parameters, leading to potential reductions in fabrication costs (energy) and lead time, as seen in Table 5.

**Table 5:** Summary of Ti-6Al-4V properties achieved using a variety of PBF process parameter combinations.

Specimen ID	WROUGHT	EOS DEF	MOD 1	MOD 3	MOD 7	MOD 9	MOD 11
**Laser scan speed (mm/s)**	n/a	1200	1000	1200	1200	500	200
**Laser power (W)**	n/a	280	280	200	120	120	80
**VED (J/mm**^**3**^**)**	n/a	56	67	40	24	57	95
**Avg. E**_**corr**_ **(mV**_**SCE**_**)**	-451 ± 70	-412 ± 23	-348 ± 39	-398 ± 76	***-158 ± 29***	-615 ± 60	-603 ± 42
**Avg. I**_**corr**_ **(**μ**A/cm**^**2**^**)**	0.07 ± 0.03	0.10 ± 0.05	***0*.*07 ± 0*.*06***	0.13 ± 0.07	1.06 ± 0.14	***0*.*12 ± 0*.*08***	0.08 ± 0.02
**Avg. R**_**a**_ **(**μ**m)**	n/a	4.62 ± 0.90	0.66 ± 0.19	***0*.*40 ± 0*.*01***	6.67 ± 1.26	***0*.*53 ± 0*.*15***	1.11 ± 0.82
**Avg. HRC**	36	48.7 ± 3.2	46.7 ± 3.5	46.7 ± 2.8	***35*.*6 ± 2*.*0***	46.9 ± 2.6	46.4 ± 1.9
**Avg. porosity (%)**	n/a	0.03 ± 0.01	0.17 ± 0.08	0.07 ± 0.01	2.48 ± 0.31	0.06 ± 0.01	***0*.*04 ± 0*.*01***
**Avg. pore area (**μ**m**^**2**^**)**	n/a	41.6 ± 3.3	***43*.*8 ± 4*.*1***	467.2 ± 35.5	2035.0 ± 133.2	286.5 ± 56.4	271.0 ± 45.3
**Comment**	Wrought Ti-6Al-4V	Manufacturers settings	Lowest I_corr,_ small pores	Lowest R_a_	Least active E_corr_, lowest hardness	Low I_corr_ and Ra	Low % porosity

Corrosion resistance must also be considered if choosing to increase surface roughness or porosity. Ti-6Al-4V normally exhibits high corrosion resistance due to its spontaneously formed passive oxides able to withstand the local bodily environment (i.e. joint fluid). PBF Ti-6Al-4V will necessarily behave differently to wrought Ti-6Al-4V, due to but not limited to the peculiarities of the melt pool and rapid solidification related solute segregation. As-built PBF Ti-6Al-4V is composed of majority α’ phase, which is reported by some groups to be less resistant to corrosion [68]. Following stress relieving procedures, the decomposition of the metastable α’ phase into lower energy α + β phases, the corrosion behaviour of PBF Ti-6Al-4V has been reported to more closely match that of wrought Ti-6Al-4V [80]. Ti-6Al-4V is generally reported to have a predominantly TiO_2_ passive layer and TiO/Ti_2_O_3_ sub-oxides, with small amounts of aluminium and vanadium oxides [81–83]. However, bulk microstructure affects surface oxide formation. Some groups have reported that the solute partitioning between α and β phases in Ti-6Al-4V (Al segregates to the α phase while V segregates to the β phase) affects surface oxide formation, with vanadium oxide dissolution in the biphasic oxide film leading to cation vacancy generation and thus weakening of the film [81,84]. The interfacial regions between the phases (both bulk and surface) are also reported to be more susceptible to corrosion [85]. Thus, it is not clear which of the α’ or mixed α+β phases are more desirable in terms of corrosion resistance. Changing the laser scan speed and/or laser power appear to have an effect on corrosion resistance and current density, not only through the characteristic changes in porosity and roughness, but also likely through changes in surface chemistry arising from keyholing or partial melting. PBF Ti-6Al-4V samples produced using laser power < 120W and laser scan speed < 1200 contained porosity > 1%, and displayed significantly narrowed repassivation windows (likely due to changes in the formation of the surface oxide), with increased corrosion current densities (>1μA/cm2). Thicker oxide layers are accepted to be more protective against corrosion [82].

Following analysis of porosity, roughness and hardness, it was determined that there was little relationship between VED and final PBF Ti-6Al-4V properties. Despite this, VED is often used in the pre-fabrication stage to predict and differentiate the final properties of PBF Ti-6Al-4V produced with differing process parameters. It is more pertinent to choose individual PBF process parameters with an understanding of how these process parameters work together to produce the final Ti-6Al-4V product. Moreover, VED as it stands describes the immediate environment of the moving laser, and so is a layer-wise descriptor at best. It is important to reduce the dependence on VED as a design variable for PBF Ti-6Al-4V.

This study was not without limitations, in large part due to the examination of top, or x-y, surfaces only. Future work should focus on curved surfaces and cross-sections for improved microstructural analysis.

The properties investigated in this study relate to the ultimate bio-activity of PBF Ti-6Al-4V. It is important to note that although the choice of parameters may require the sacrifice of certain features (i.e. lower porosity but higher corrosion resistance), implants may well be fabricated through PBF in two parts–the bulk vs. the shell. The properties of the shell, or outer layers, of the implant are the most important as they directly impact osseointegration and thus the achievement of successful long-term implant fixation.

## Conclusions

Powder Bed Fusion has the potential to produce effective and long-lasting customised Ti-6Al-4V orthopaedic implants, with the careful control of process parameters used during fabrication. Key findings regarding the effect of varying laser scan speed and laser power on PBF Ti-6Al-4V were as follows;

- The EOS DEF settings for Ti-6Al-4V (280 W, 1200 mm/s, VED = 55 J/mm^3^) produced samples with 0.03 ± 0.01% porosity, irregular pore shapes, R_a_ of 4.6 ± 0.9 μm, E_corr_ of -412 ± 23 mV and hardness of 48.7 ± 3.2 HRC.
- Each of the other process parameter combinations produced samples with one or more improved properties when compared to wrought Ti-6Al-4V, and so it is clear that sample properties can be tuned through careful manipulation of the PBF process parameters, leading to potential reductions in fabrication costs (energy) and lead time, as seen in Table 5.
- Changes in laser scan speed and laser power affect melt pool formation; the dynamic equilibrium of the melt pool is affected by surface tension and thermo-capillary convection flow. Increasing laser scan speed produces elongated melt pools with increased Plateau-Rayleigh instabilities, and higher likelihood of balling. Increasing laser power deepens the melt pool and increases the number of remelting/recooling cycles experienced by each layer. Laser scan speed and laser power must be balanced to ensure full powder consolidation.
- The combination of high laser scan speed (> 1000 mm/s) with low laser power (< 120 W) in the PBF fabrication of Ti-6Al-4V leads to poor powder consolidation due to the insufficient supply of thermal energy per unit volume. This results in porosity levels of above 15%, high roughness, low hardness and increased susceptibility to corrosion.
- The PBF fabrication of Ti-6Al-4V with low laser scan speed (< 200 mm/s) and high laser power (> 100W) results in the supply of excessive thermal energy per unit volume, and more specifically, increases in layer wise laser penetration depth and keyholing.
- The combination of intermediate laser power and laser scan speed produces the most homogeneous PBF Ti-6Al-4V surfaces, with low amounts of evenly distributed pores, low roughness and hardness/corrosion resistance similar to conventionally produced Ti-6Al-4V.

Presently, there is little consensus in the biomedical community on the optimal porosity/roughness for osseointegration [74–76]; however once osseointegration is better understood, the results of this study can be used to choose PBF process parameters to produce Ti-6Al-4V implants with the required properties. For example, if osseointegration with Ti-6Al-4V implants is definitively found to be enhanced when surfaces have a minimum R_a_ of 0.5 μm and maximum porosity of 0.7%, then it is clear that a laser scan speed of 200 mm/s and laser power of 280 W could be used.

The present study examined the effects of altering laser scan speed and laser power on PBF Ti-6Al-4V, and provides a map of the PBF process with respect to achievable properties relevant to biomedical applications. The most consistent properties are observed in samples built with ‘balanced’ laser scan speed and laser power, i.e. MODS 9 (120 W, 500 mm/s), 11 (80 W, 200 mm/s), 16 (200 W, 500 mm/s), 20 (120 W, 1000 mm/s) and EOS DEF (280 W, 1200 mm/s). These parameters however are not the only ones which affect final component properties–other important PBF process parameters include spot size, scan strategy, particle characteristics, and temperature uniformity, as well as second order factors include defocussing distance, gas shielding and time between passes [86]. Studies investigating the interplay of these additional PBF process parameters are required to build on the relationships observed in the current study.

## Supporting information

S1 Fig X-ray diffraction data for SLM Ti-6Al-4V produced with various process parameter combinations, with all specimens displaying peaks corresponding to tetragonal Ti-6Al-4V (PDF Ref. 01-072-5007), hexagonal close packed α’ phase Ti (PDF ref. 00-044-1294), and cubic V (PDF Ref. 00-022-1058) and none corresponding to body centred cubic β-Ti (PDF Ref. 00-044-1288). (TIF)

S1 Table Summary of data collected/determined for SLM Ti-6Al-4V specimens fabricated using varying combinations of laser power and laser scan speed. (TIF)

S2 Table Major components of Minimum Essential Media formulation (Thermofisher #61100) used as electrolyte in electrochemical testing. **(full composition available at (http://www.thermofisher.com/au/en/home/technical-resources/media-formulation.109.html))** [87].
(TIF)
