# Supplementary figures and images for: Understanding the effects of PBF process parameter interplay on Ti-6Al-4V surface properties
Source: PLoS One. 2019 Aug 29;14(8):e0221198. doi: 10.1371/journal.pone.0221198 (PMC6715245; doi:10.1371/journal.pone.0221198)

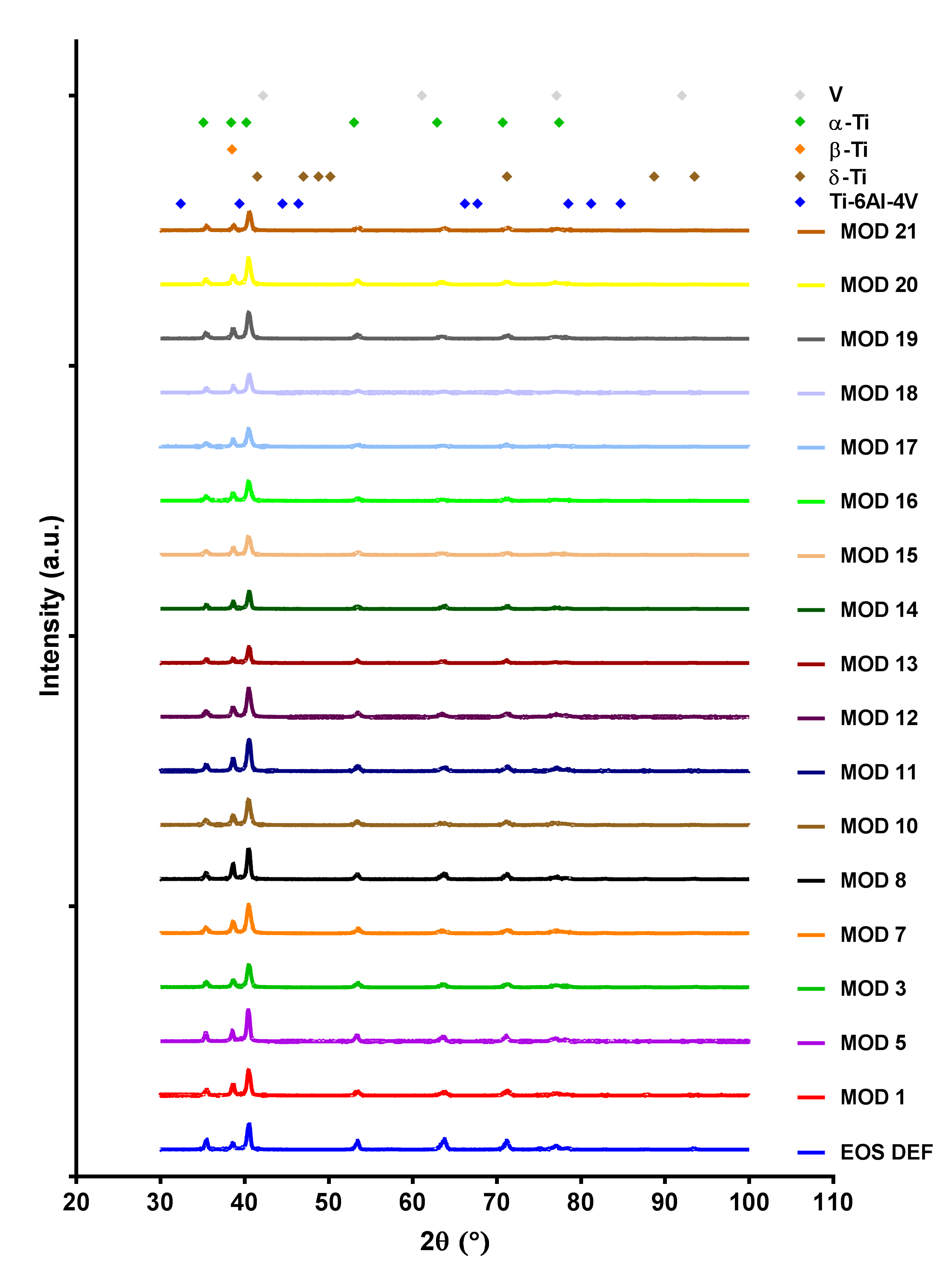

Supplement: S1 Fig — (TIF) [file pone.0221198.s001.tif]

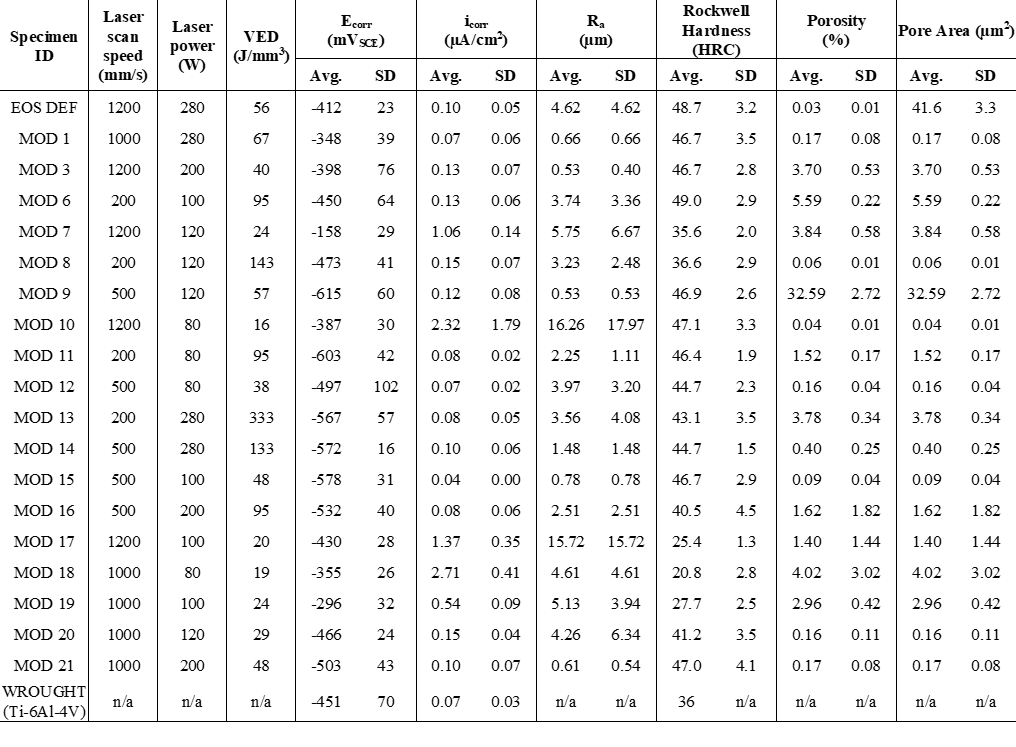

Supplement: S1 Table — (TIF) [file pone.0221198.s002.tif]

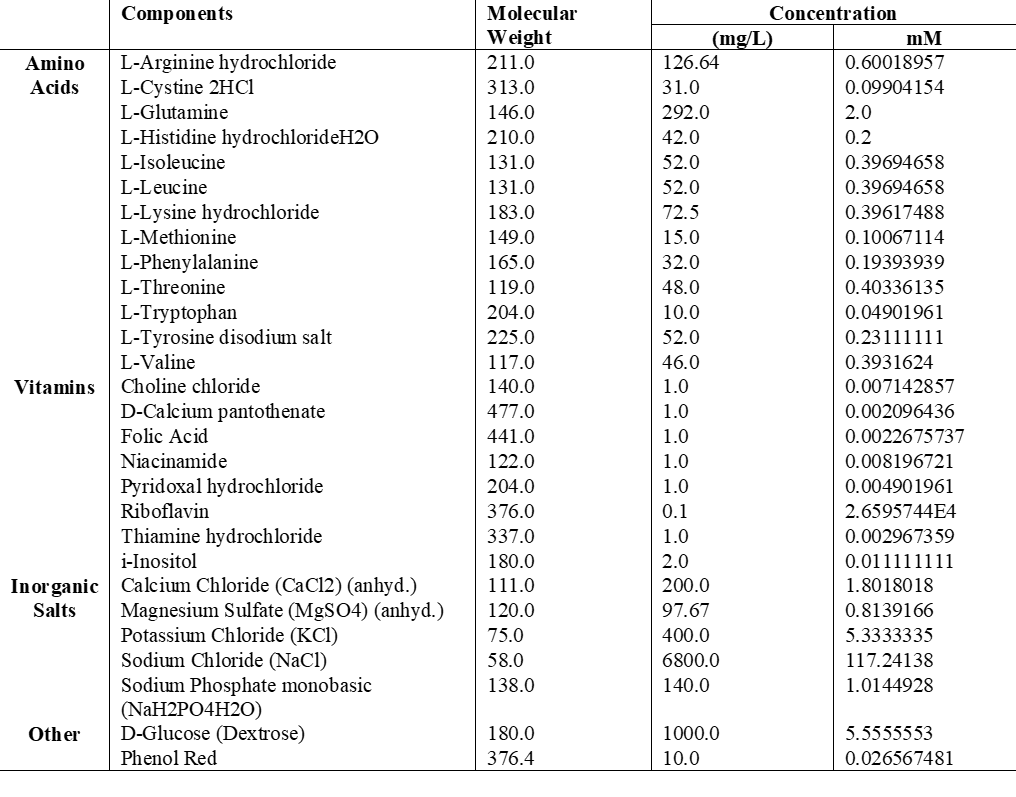

Supplement: S2 Table — (full composition available at (http://www.thermofisher.com/au/en/home/technical-resources/media-formulation.109.html)) [87]. (TIF) [file pone.0221198.s003.tif]
